# Investigation on the Efficiency of Tonic Chinese Herbal Injections for Treating Dilated Cardiomyopathy Based on Bayesian Network Meta-Analysis

**DOI:** 10.1155/2021/8838826

**Published:** 2021-04-01

**Authors:** Kaihuan Wang, Haojia Wang, Jiarui Wu, Xiaojiao Duan, Xinkui Liu, Dan Zhang, Shuyu Liu, Mengwei Ni, Ziqi Meng, Xiaomeng Zhang

**Affiliations:** Department of Clinical Chinese Pharmacy, School of Chinese Materia Medica, Beijing University of Chinese Medicine, Beijing 100029, China

## Abstract

**Introduction:**

This network meta-analysis investigated the efficacy of six tonic Chinese herbal injections (Huangqi injection, Shenfu injection, Shengmai injection, Shenmai injection, Shenqi Fuzheng injection, and Yiqifumai injection) compared to Western medicine for the treatment of the deteriorating state associated with dilated cardiomyopathy.

**Methods:**

PubMed, the Cochrane Library, Embase, the Chinese Biological Medicine Database, China National Knowledge Infrastructure, the Wanfang Database, and the Chinese Scientific Journal Database were searched from their inception to October 15, 2020, to retrieve randomized controlled trials (RCTs). Study selection and data extraction conformed to a priori criteria. The risk of bias of the included RCTs was determined, and GRADE was used to evaluate outcomes. The network meta-analysis was calculated using WinBUGS 1.4.3 and Stata 13.0 software. The clinical effective rate, left ventricular ejection fraction, 6-minute walk test, left ventricular end-diastolic dimension, heart rate, and cardiac output were deemed outcomes. All outcomes were summarized as odds ratios or mean differences with their 95% credible intervals. The ranking probability of the interventions across various outcomes was also presented.

**Results:**

Forty RCTs and 2970 patients were enrolled. Integration of the outcome results revealed that a combination of Shenfu injection and Western medicine ranked ahead of the other injections in most outcomes, especially in the clinical effective rate (OR = 0.21, 95% CI: 0.12–0.34), left ventricular ejection fraction (MD = 7.43, 95% CI: 2.41–12.38), and 6-minute walk test (MD = 50.39, 95% CI: 25.78–76.33). Shenmai injection plus Western medicine ranked ahead of the other injections in left ventricular end-diastolic dimension (69.5%) and cardiac output (60.9%). The cluster analysis suggested that Shenfu injection plus Western medicine was the most effective intervention for dilated cardiomyopathy.

**Conclusions:**

Shenfu injection plus Western medicine may be a preferable treatment in dilated cardiomyopathy. Clinicians should also consider the specific patient's various conditions when making diagnostic decisions. Due to an insufficient network meta-analysis, more high-quality RCTs need to be implemented to support our conclusions.

## 1. Introduction

Dilated cardiomyopathy (DCM) is the most common cardiomyopathy worldwide, and it refers to a heart muscle disease that is characterized by left ventricular and biventricular dilatation and systolic dysfunction without volume overload or coronary artery disease. These conditions result in decreased cardiac output and stroke volume, increased end-diastolic pressure, and ultimately malignant arrhythmia, heart failure, and even sudden death [1–4]. DCM patients suffer from a lower quality of life and heavy economic pressure with a high hospitalization rate and mortality [1, 5]. The estimated prevalence of DCM was 40 cases in 100,000 individuals with an annual incidence of 7 cases in 100,000 individuals [1, 2]. Several factors may contribute to DCM, such as persistent infection, autoimmunity, gene mutation, and genetic factors [4, 6, 7]. The mechanisms underlying the deterioration in cardiac function are largely unknown [4, 6–8], but therapies in most cases concentrated on the symptoms of heart failure and related complications [4, 6, 7]. Angiotensin-converting enzyme inhibitors, *β* blockers, digoxin, and diuretics are standard treatment options.

With the promotion of traditional Chinese medicine, its utilization is becoming increasingly instrumental to the treatment of DCM. Traditional Chinese medicine theories consider DCM a “heart impediment,” “chest obstruction,” and “edema” that emerges in the heart and then affects the lung, spleen, and kidney [7, 9]. The clinical principle is primarily directed at strengthening the body's resistance to eliminate pathogenic factors. As an indispensable part of traditional Chinese medicine, Chinese herbal injections (CHIs) are vital for the treatment of DCM with Western medicine (WM) treatment [10–16]. Tonic CHIs refer to injections whose main components are ginseng, astragalus, and *Ophiopogon japonicus*, which have the functions of replenishing qi, nourishing blood, nourishing yin, and assisting yang, and are primarily used to treat various deficiency symptoms. The present network meta-analysis (NMA) incorporated six common tonic CHIs that are used in the treatment of DCM, namely, Huangqi injection, Shenfu injection, Shengmai injection, Shenmai injection, Shenqi Fuzheng injection, and Yiqifumai injection, to determine their efficacy. The China Food and Drug Administration authorized all of these tonic CHIs.

We retrieved the relevant RCTs, and most of the clinical trials compared the efficacy between one of the aforementioned six tonic CHIs plus WM and WM alone rather than a head-to-head comparison of the CHIs. NMA is a technique that allows researchers to meta-analyse more than two interventions simultaneously according to a combination of direct and indirect evidence [16–18]. We performed an NMA to provide an overview of the efficacy of tonic CHIs and more guidance in the selection of DCM treatment.

## 2. Methods

This NMA was performed according to the PRISMA Extension Statement for Reporting of Systematic Reviews Incorporating Network Meta-analyses of Health Care Interventions [19]. A completed PRISMA checklist is included in the supplementary material (available here) (PRISMA Checklist).

### 2.1. Data Sources and Searches

The included studies were acquired after comprehensive database searches of PubMed, the Cochrane Library, Embase, the Chinese Biological Medicine Database, China National Knowledge Infrastructure, the Wanfang Database, and the Chinese Scientific Journal Database from their inception to October 15, 2020. Additional relevant studies were retrieved from the reference lists of previous meta-analyses and the included studies to avoid omission. For example, two reviewers developed the following search strategy in PubMed: (huangqi [Title/Abstract] OR astragalus [Title/Abstract] OR shenfu [Title/Abstract] OR shengmai [Title/Abstract] OR shenmai [Title/Abstract] OR shenqi fuzheng [Title/Abstract] OR yiqifumai [Title/Abstract]) AND (dilated cardiomyopathy [MeSH terms] OR dilated cardiomyopathy [Title/Abstract]) AND (randomized controlled trial [Publication Type] OR controlled clinical trial [Publication Type] OR randomized [Title/Abstract] OR placebo [Title/Abstract] OR clinical trials as topic [MeSH Major Topic] OR randomly [Title/Abstract] OR trial [Title/Abstract]) More details on the strategy of CHIs are provided in the supplementary material (available here).

### 2.2. Eligibility Criteria

This NMA included randomized controlled trials (RCTs) that reported the efficacy of six CHIs combined with WM for treating DCM. The predesigned study inclusion criteria for this NMA were as follows: (1) RCTs with a minimum sample size of 15 patients in each group. No limitations on language, publication year, or publication status were applied. (2) The patients were diagnosed with DCM according to standard diagnostic criteria. No restrictions on gender, race, and nationality were applied. (3) The interventions met the standard framework of “CHI + WM versus WM” or “CHI + WM versus CHI + WM.” WM consisted of angiotensin-converting enzyme inhibitors, *β*-blockers, diuretics, and cardiotonics. Patients received other relevant therapies if they had complications during the therapeutic process. (4) The RCTs reported the clinical effective rate or left ventricular ejection fraction. Cardiac function classification conformed to the standard issued by the New York Heart Association in the United States. The clinical effective rate was calculated using this formula: (number of remarkable recovery patients + number of basic recovery patients)/total number of patients *∗* 100%. Patients in whom clinical symptoms disappeared and cardiac function improved at least 2 levels were classified as remarkable recovery, and patients with relieved clinical symptoms and an increase in cardiac function of 1 level were regarded as basic recovery. If patients' clinical symptoms and cardiac function were unaltered or worse, they were classified as patients showing deterioration. The aforementioned outcomes were set as primary outcomes, and the 6-minute walk test, left ventricular end-diastolic dimension, heart rate, cardiac output, and adverse drug reactions/adverse drug events (ADRs/ADEs) measures were evaluated as secondary outcomes. (5) The full text of the RCTs could be assessed.

### 2.3. Study Selection and Data Extraction

Two reviewers screened the title, abstract, and keywords of every retrieved study to determine which studies required further assessment. The full text of potential studies was examined in detail. In cases of disagreement between reviewers, a consensus was obtained via discussion or consultation with a third reviewer. A standardized data extraction form was designed using Microsoft Excel 2016 to collect data from the included RCTs. The data extraction items included the first author's name, publication year, country, patient characteristics (sample size, gender, age, patients' baseline cardiac function classification, and disease duration), details of the intervention, outcomes, the RCT design, and the domains for a risk of bias assessment.

### 2.4. Quality Assessment

The Cochrane Collaboration risk of bias tool was used to evaluate the risk of bias of the included RCTs. The evaluation items included sequence generation (selection bias), allocation concealment (selection bias), blinding of patients and personnel (performance bias), blinding of outcome assessors (detection bias), incomplete outcome data (attrition bias), selective reporting (reporting bias), and other biases. Each item was appraised as “low risk,” “unclear risk,” or “high risk” as a description for the included RCTs. Any conflicts were resolved via discussion or consultation.

Based on the results of the systematic review and network meta-analysis, this study adopted GRADE (Grading of Recommendations Assessment, Development, and Evaluation) to evaluate the evidence quality of outcomes. In GRADE system, RCTs are set as the highest level of evidence at the starting point, and its down rating indicators include limitations (risk of bias), inconsistency, indirectness, imprecision, and publication bias. Up rating indicators are only applied to observational studies, which include effect size, confounding factors, and dose-effect relationship.

### 2.5. Data Analysis

This NMA was performed for each outcome in a Bayesian hierarchical framework using WinBUGS 1.4.3 software (MRC Biostatistics Unit, Cambridge, UK). Other correlative graphical representations were processed using Stata 13.0 software. The random effects model in WinBUGS was selected. The number of iterations was set to 200,000, and the first 10,000 were used for the annealing algorithm to eliminate the impact of the initial value. For binary outcomes, an odds ratio (OR) with a 95% credible interval (95% CI) was produced. A mean difference (MD) with its 95% CI was generated for continuous outcomes. A 95% CI of OR that did not include 1.00 or a 95% CI of MD that did not include 0.00 was considered statistically significant.

To obtain the rank of each intervention for the various outcomes, this NMA calculated the surface under the cumulative ranking (SUCRA) value to estimate the ranking probabilities for each intervention. The SUCRA value was interpreted as a percentage. The SUCRA value of the best intervention was 100%. Otherwise, the SUCRA value was 0% [20, 21]. To determine the most efficacious injection in treating DCM, a cluster analysis for primary outcomes was performed [20, 22]. The consistency of direct and indirect evidence is an essential assumption of NMA [23, 24]. Therefore, if the closed loop of interventions was available, a loop-specific approach was examined to check the inconsistency of evidence. An inconsistency factor and its 95% CI was calculated to estimate the presence of inconsistency in each loop. It was deemed a consistency between direct and indirect evidence when *p* > 0.05 [25]. A funnel plot was also created to assess the publication bias [26, 27]. The network graph, the forest plot, and the contribution plot are also depicted [26, 27].

This NMA depended on previously published studies. As a result, ethical approval was not necessary.

## 3. Results

### 3.1. Study Selection

**Figure 1:**
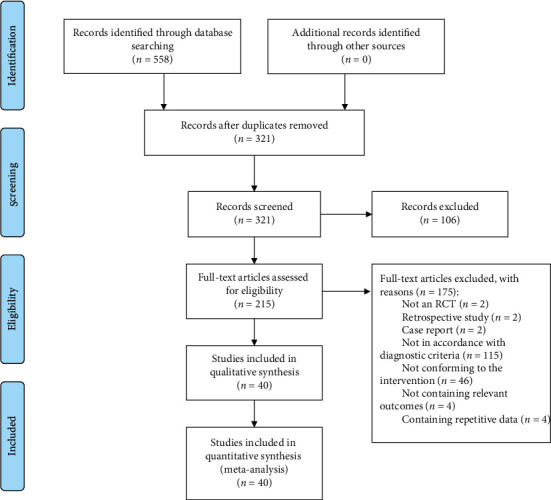
PRISMA flow diagram (*n*, number of articles).

Of 558 potential studies scanned, 215 studies were further assessed. From these studies, 175 studies were excluded for the following reasons: (1) was not an RCT (*n* = 2); (2) was a retrospective study (*n* = 2); (3) was a case report (*n* = 2); (4) was not in accordance with the diagnostic criteria (*n* = 115); (5) did not conform to the requirements of the intervention (*n* = 46); (6) did not contain the relevant outcomes (*n* = 4); (7) contained repetitive data (*n* = 4). Forty-two-armed RCTs were ultimately included and analysed. All included RCTs were published and implemented in China from 1998 to 2017 (Figure 1).

### 3.2. Study Characteristics and Quality Evaluation

**Figure 2:**
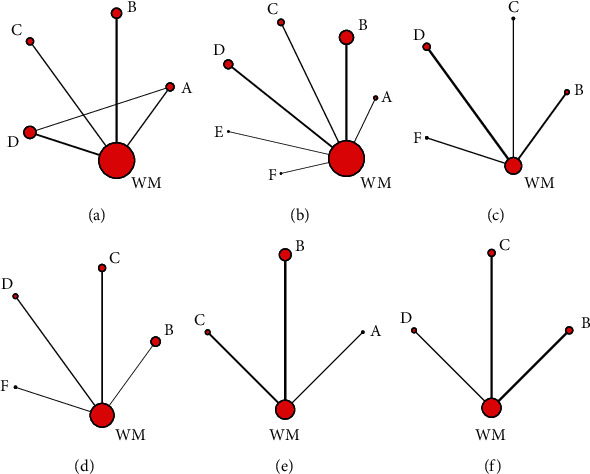
Network graphs for different outcomes. A, Huangqi injection + WM; B, Shenfu injection + WM; C, Shengmai injection + WM; D, Shenmai injection + WM; E, Shenqi Fuzheng injection + WM; F, Yiqifumai injection + WM. (a) The clinical effective rate. (b) Left ventricular ejection fraction. (c) 6-minute walk test. (d) Left ventricular end-diastolic dimension. (e) Heart rate. (f) Cardiac output.

A total of 2970 patients and six CHIs were included, including 1503 patients in the treatment group and 1467 patients in the control group. Male patients were approximately 59.5% of the total, and the age of patients ranged from 20 to 81 years. Thirty-nine included RCTs compared the efficacy between one of the included CHIs plus WM and WM, and 1 RCT compared Shenmai injection plus WM with Huangqi injection plus WM. WM included angiotensin-converting enzyme inhibitors, *β*-blockers, cardiotonics, and diuretics. CHIs were used in the following proportions in the 40 RCTs: Huangqi injection (7 RCTs), Shenfu injection (12 RCTs), Shengmai injection (7 RCTs), Shenmai injection (12 RCTs), Shenqi Fuzheng injection (1 RCT), and Yiqifumai injection (1 RCT). All included CHIs were intravenously injected. Patients in most RCTs received CHIs once a day, and patients in only 1 RCT received a CHI twice a day. The treatment course in most trials was 14 days. According to outcomes, 90% of the RCTs reported the clinical effective rate, and 70% of RCTs investigated the left ventricular ejection fraction. Six RCTs adopted the dialectical theory based on traditional Chinese medicine in the treatment of DCM. The main characteristics of the included RCTs are shown in Table 1, and the network graphs for various outcomes are illustrated in Figure 2.

**Figure 3:**
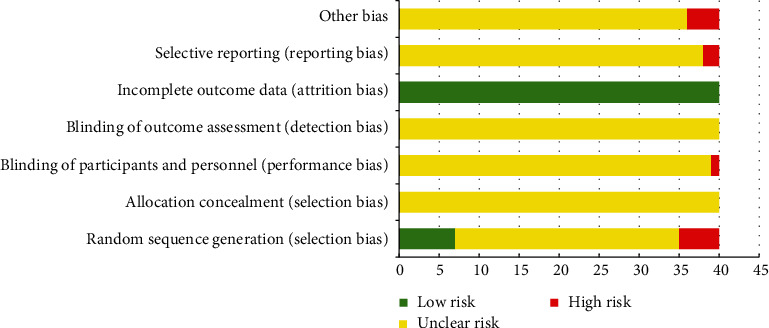
Assessment of risk bias.

Overall, the quality of the included RCTs was general. For selection bias, 7 RCTs were deemed “low risk” because they used a random number table method to generate the sequence. Five RCTs were deemed “high risk” because they generated the sequence using some rule based on hospital or date of admission. Only 1 RCT used a single-blind method and was evaluated as “high risk” in performance bias because the colour and usage of CHIs suggested the formulation. All included RCTs reported complete outcome data and were subsequently evaluated as “low risk” in attrition bias. Two RCTs did not report all outcomes in its design and were deemed “high risk” in reporting bias. Four RCTs did not refer to whether there was comparability between the treatment group and the control group, which may broaden the difference between the results and influence the outcome data. Therefore, these 4 RCTs were assessed as “high risk” in other biases. The remaining items were considered “unclear risk” due to insufficient information (Figure 3).

The four outcomes including the clinical effective rate, left ventricular ejection fraction, 6-minute walk test, and left ventricular end-diastolic dimension were evaluated by GRADE classification. The other two outcomes were not graded due to the insufficient amount of literature included in their comparison. We came to the following conclusions. (1) In the NMA results of the clinical effective rate, HQI + WM vs WM, SFI + WM vs WM, SMI + WM vs WM, and HQI + WM vs SMI + WM were evaluated as low quality, and other comparisons were evaluated as very low. (2) In the results of left ventricular ejection fraction, only the comparison of YQFMI + WM vs WM was rated as low quality, and others were very low. (3) In the results of the 6-minute walk test, the comparison of SFI + WM vs WM and SI + WM vs WM was rated as medium. And the low quality was rated for SFI + WM vs SI + WM, SFI + WM vs YQFMI + WM, SI + WM vs YQFMI + WM, and YQFMI + WM vs WM. The other comparisons were rated as very low. (4) In the rating results of the left ventricular end-diastolic dimension, SFI + WM vs SMI + WM, SFI + WM vs YQFMI + WM, SFI + WM vs WM, SMI + WM vs YQFMI + WM, SMI + WM vs WM, and YQFMI + WM vs WM were all rated as low, and the rest were rated as very low. Detailed information about the result of GRADE is shown in supplementary material (available here).

### 3.3. Outcomes

#### 3.3.1. The Clinical Effective Rate

**Figure 4:**
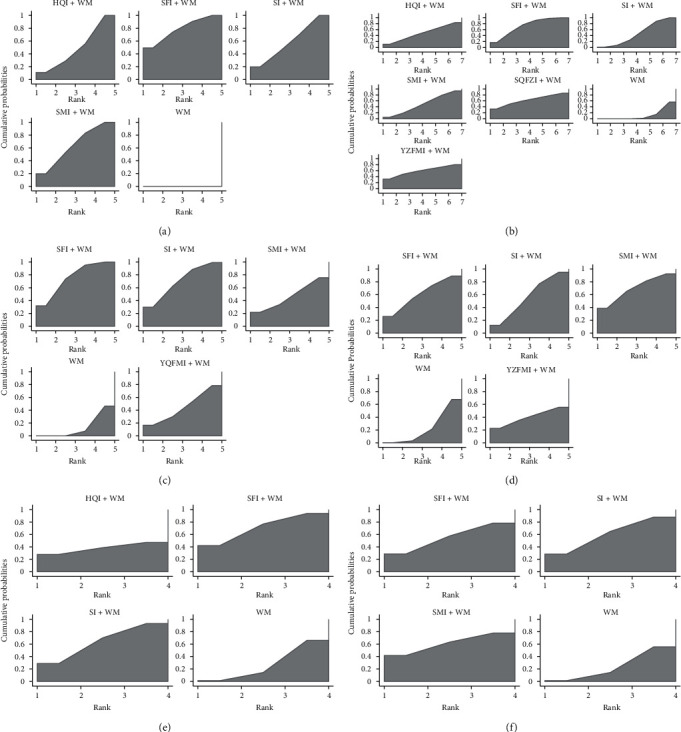
Plot of the surface under the cumulative ranking curves for all interventions for different outcomes. (a) The clinical effective rate. (b) Left ventricular ejection fraction. (c) 6-minute walk test. (d) Left ventricular end-diastolic dimension. (e) Heart rate. (f) Cardiac output.

**Figure 5:**
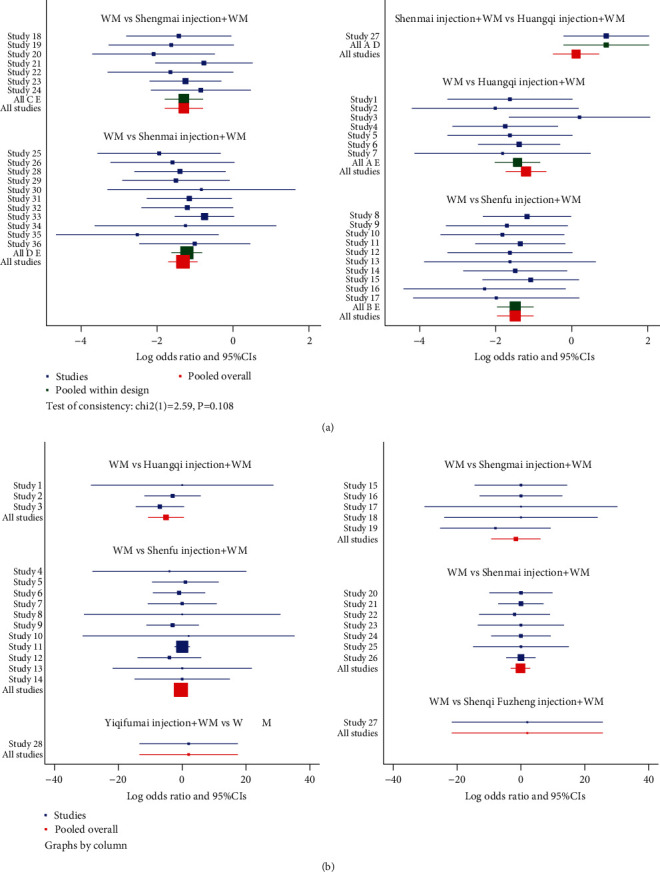
Network forest plot for the clinical effective rate and left ventricular ejection fraction. (a) The clinical effective rate. (b) Left ventricular ejection fraction.

**Figure 6:**
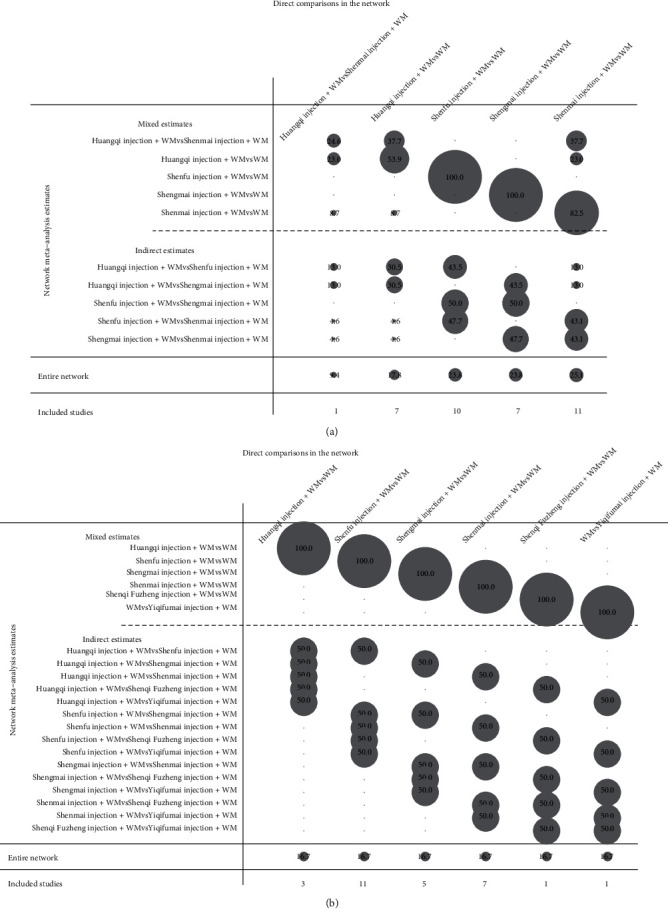
Network contribution plot for the clinical effective rate and left ventricular ejection fraction. (a) The clinical effective rate. (b) Left ventricular ejection fraction.

The clinical effective rate was the primary outcome because it directly reflects the efficacy. There were 36 RCTs that investigated this outcome (Huangqi injection, 7 RCTs; Shenfu injection, 10 RCTs; Shengmai injection, 7 RCTs; and Shenmai injection, 12 RCTs). As shown in Table 2, all included CHIs were associated with a significantly higher improvement in the clinical effective rate. Huangqi injection + WM (OR = 0.28, 95% CI: 0.16–0.48), Shenfu injection + WM (OR = 0.21, 95% CI: 0.12–0.34), Shengmai injection + WM (OR = 0.26, 95% CI: 0.15–0.43), and Shenmai injection + WM (OR = 0.24, 95% CI: 0.16–0.37) were more effective in promoting the clinical effective rate compared to WM and had statistically significant differences. The ranking analysis suggested that Shenfu injection + WM was more efficacious for the clinical effective rate with a probability of 78.5% (Table 3). Other beneficial interventions were Shenmai injection + WM (64.0%) and Shengmai injection + WM (58.5%) (Figure 4). The forest plot and the contribution plot for the clinical effective rate are shown in Figures 5 and 6.

#### 3.3.2. Left Ventricular Ejection Fraction

The left ventricular ejection fraction was also deemed a primary outcome. DCM is often accompanied by heart failure, and the left ventricular ejection fraction is a diagnostic index of heart failure and a reflection of patient prognosis [3]. Twenty-eight RCTs identified the left ventricular ejection fraction (Huangqi injection, 3 RCTs; Shenfu injection, 11 RCTs; Shengmai injection, 5 RCTs; Shenmai injection, 7 RCTs; Shenqi Fuzheng injection, 1 RCT; and Yiqifumai injection, 1 RCT). Shenfu injection + WM (MD = 7.43, 95% CI: 2.41–12.38) and Shengmai injection + WM (MD = 3.88, 95% CI: 1.10–8.05) were associated with a significantly higher mean difference in left ventricular ejection fraction than WM (Table 2). Based on its SUCRA, Shenfu injection + WM was the best in increasing left ventricular ejection fraction with a probability of 71.9%, Shenqi Fuzheng injection + WM (62.5%) was ranked second, and Yiqifumai injection + WM (59.7%) was ranked third (Table 3, Figure 4). The forest plot and the contribution plot for the left ventricular ejection fraction are shown in Figures 5 and 6.

#### 3.3.3. Secondary Outcomes

Data on the 6-minute walk test were available from 7 RCTs (Shenfu injection, 2 RCTs; Shengmai injection, 1 RCT; Shenmai injection, 3 RCTs; and Yiqifumai injection, 1 RCT). The MD results demonstrated that Shenfu injection + WM (MD = 50.39, 95% CI: 25.78–76.33) and Shengmai injection + WM (MD = 46.43, 95% CI: 5.27–88.48) had statistically significant differences compared to WM in the 6-minute walk test (Table 2). The ranking analysis indicated that Shenfu injection + WM was the most favourable intervention in promoting a 6-minute walk test with a probability of 75.2% (Table 3, Figure 4).

Data on the left ventricular end-diastolic dimension were available from 10 RCTs (Shenfu injection, 4 RCTs; Shengmai injection, 3 RCTs; Shenmai injection, 2 RCTs; and Yiqifumai injection, 1 RCT). The MD results suggested no statistically significant decreases between the included interventions for the left ventricular end-diastolic dimension (Table 2). The ranking analysis demonstrated that Shenmai injection + WM was best at decreasing left ventricular end-diastolic dimension with a probability of 69.5% (Table 3, Figure 4). A total of 8 RCTs that included three CHIs (Huangqi injection, 1 RCT; Shenfu injection, 5 RCTs; and Shengmai injection, 2 RCTs) contributed to the analysis of heart rate. No significant differences were observed between the various interventions (Table 2). Based on the ranking analysis, Shenfu injection + WM attained the highest rank in heart rate relief with a probability of 70.9% (Table 3, Figure 4). Eight RCTs also tested cardiac output (Shenfu injection, 3 RCTs; Shengmai injection, 3 RCTs; and Shenmai injection, 2 RCTs). None of the included interventions produced significant improvements in cardiac output (Table 2). The ranking analysis showed that Shenmai injection + WM had the best impact on boosting cardiac output with a probability of 60.9% (Table 3, Figure 4).

#### 3.3.4. Cluster Analysis and Radar Presentation

**Figure 7:**
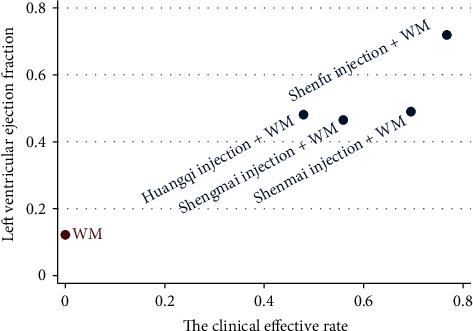
Cluster analysis plot for four outcomes.

A cluster analysis was performed for the primary outcomes to evaluate the best intervention for the treatment of DCM. Shenfu injection + WM was the farthest from zero relative to the other interventions, which suggested that it was more effective than the other treatments for DCM (Figure 7).

**Figure 8:**
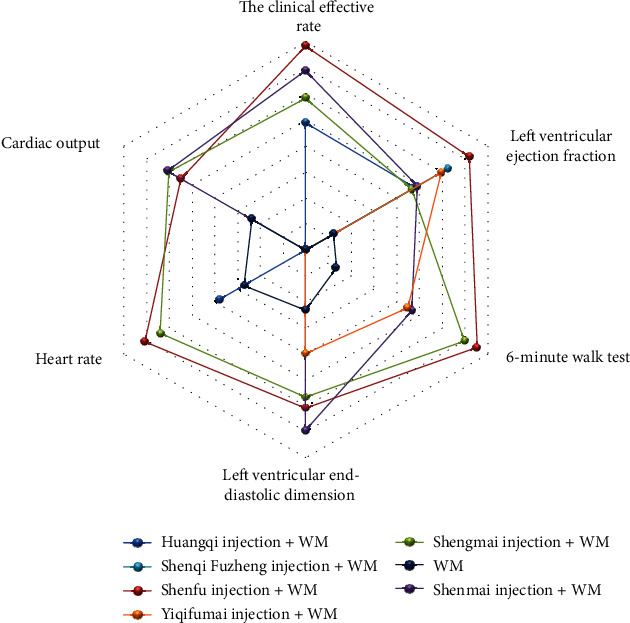
Radar map of different outcomes.

As a way to synthesize the SUCRA results of the various interventions across outcomes, this NMA created a pictorial presentation for the outcomes via a radar map. If the injection exhibited outstanding efficacy relative to other treatments for a certain outcome, it would appear on the outermost side of the corresponding line in the radar map.  Figure 8 shows that the Shenfu injection excelled at increasing the clinical effective rate, left ventricular ejection, 6-minute walk test, and calming heart rate.

#### 3.3.5. Publication Bias

**Figure 9:**
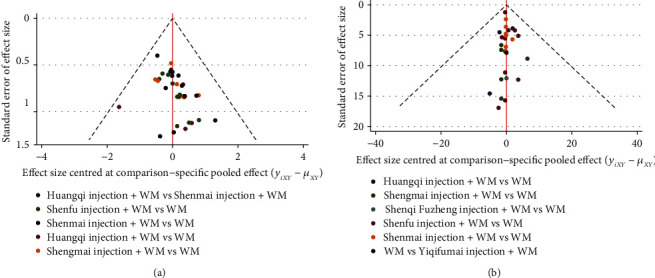
Funnel plot for the clinical effective rate and left ventricular ejection fraction. (a) The clinical effective rate. (b) Left ventricular ejection fraction.

Publication bias was detected using funnel plots for the primary outcomes. Visual inspection showed asymmetry in the clinical effective rate, but the funnel plot of the left ventricular ejection fraction did not display any asymmetry (Figure 9).

#### 3.3.6. Inconsistency Test

Loop-specific analysis did not find any inconsistency in the clinical effective rate. The *p*-value of the loop of Huangqi injection + WM-Shenmai injection + WM-WM was above 0.05, and the inconsistency factor was 1.072 (0.00, 2.41), which determined that the direct and indirect evidence were consistent.

#### 3.3.7. ADRs/ADEs

Nine RCTs (Huangqi injection, 2 RCTs; Shenfu injection, 1 RCT; Shengmai injection, 1 RCT; and Shenmai injection, 5 RCTs) reported that there were no ADRs/ADEs during the treatment, and 3 RCTs reported that ADRs/ADEs had occurred. The remaining RCTs did not report ADRs/ADEs in their publication. The interventions resulting in the ADRs/ADEs in the 3 RCTs were the control group WM in one RCT and the treatment group Shengmai injection + WM in two RCTs. In Li's study [28], 1 case of hypotension occurred in the treatment group, and 7 cases of hypotension and palpitation occurred in the corresponding control group. In Shi's research [29], the treatment group experienced 2 cases of rash, 3 cases of insomnia, 2 cases of arrhythmia, and 1 case of flush, and the corresponding control group experienced 1 case of rash, 2 cases of insomnia, 2 cases of arrhythmia, and 1 case of flush. One RCT [30] reported 2 cases of mild gastrointestinal reaction in the Shenmai injection + WM treatment group. All of the symptoms were treated and did not affect the results.

## 4. Discussion

DCM has received increasing attention due to its prevalence and high mortality, and with the adoption of tonic CHIs in the treatment of DCM based on WM, its efficacy has been perfected. Traditional Chinese medicine theory generally believes that the cause of DCM is congenital deficiency and acquired disorders. With the pathogenesis of “ben xu biao shi,” i.e., low physical fitness and severe disease symptoms, it is necessary to use WM to relieve symptoms and use tonic drugs to regulate the foundation of the innate condition. Although most RCTs and pairwise meta-analyses investigated CHIs' efficacy, the number of RCTs that directly compared tonic CHI treatments was insufficient. Based on this lack of studies, clinicians cannot acquire an overview of the efficacy of various tonic CHIs. However, an NMA may address this void because it is useful to have comparative efficacies in the absence of head-to-head comparisons. The ranking analysis of the CHIs in the treatment of DCM was verified [31, 32]. We performed an NMA to examine the efficacy of these seven interventions: Huangqi injection + WM, Shenfu injection + WM, Shengmai injection + WM, Shenmai injection + WM, Shenqi Fuzheng injection + WM, Yiqifumai injection + WM, and WM.

There were three principal findings that provided new evidence on the efficacy of tonic CHIs for the treatment of DCM: (1) relative to other treatments, Shenfu injection + WM resulted in a significantly greater increase in the clinical effective rate, left ventricular ejection fraction, and 6-minute walk test; (2) with respect to left ventricular end-diastolic dimension, heart rate, and cardiac output, Huangqi injection + WM, Shenfu injection + WM, Shengmai injection + WM, Shenmai injection + WM, Shenqi Fuzheng injection + WM, and Yiqifumai injection + WM presented no significant differences between treatments and had a good impact on these outcomes; and (3) the safety of the included CHIs could not be ascertained due to insufficient information. Although most comparisons revealed no significant differences in the NMA, the SUCRA values demonstrated the ranking probability of the included interventions. Shenfu injection + WM obtained a high probability of becoming the most efficacious intervention for the clinical effective rate, left ventricular ejection fraction, 6-minute walk test, and heart rate. Shenmai injection + WM ranked ahead of the other treatments in left ventricular end-diastolic dimension and cardiac output. Based on the SUCRA value, the cluster analysis suggested that Shenfu injection + WM was the most effective intervention for DCM. Shenfu injections stemmed from Shenfu Tang, which is generally used for cardiovascular diseases in China [33]. Its major ingredients are the extracts of Ginseng Radix et Rhizoma Rubra and Aconm Lateralis Radix Praeparata, and Shenfu injections tonify qi, restore yang, and prevent exhaustion [34]. Pharmacological research revealed that Shenfu injection had a positive inotropic effect [35]. It promotes ventricle contractility, reduces blood viscosity, and optimises myocardium blood flow [33]. Several pairwise meta-analyses showed that Shenfu injection plus WM exhibited a superior capability to increase efficacy in a 6-minute walk test and a lower left ventricular end-diastolic dimension [12].

The safety of the interventions is also an essential consideration for clinicians. Approximately two-thirds of the included RCTs did not report ADRs/ADEs, which meant clinicians paid less attention to this aspect. However, CHIs are delivered via intravenous administration, and their ADR/ADE risk is higher than other administration routes [36]. Therefore, it is the responsibility of the clinicians to focus on its safety, and particularly on its dose and the solution [37]. In this NMA, three of the included RCTs exceeded the specified dose, and 2 RCTs injected CHIs based on the patients' weight, which may have also exceeded the regulated dose. Only 1 of these RCTs showed no ADRs/ADEs in the treatment process. However, the remaining RCTs did not mention the safety situation due to lower concentrations. The Huangqi injection specification did not mention the solution, and Shenqi Fuzheng injection did not need a solution. The remaining CHIs in the included RCTs abided by the specifications. Although most RCTs used solutions that conformed to the specification, it is necessary to provide a more detailed description. For example, the specification should state whether 0.9% sodium chloride or insulin was used for diabetes.

Based on the design and contents, three merits enhanced the credibility of this NMA. First, this NMA based on a Bayesian analysis included a comprehensive literature search and examined the comparative efficacy of six CHIs for the first time. The Bayesian model was the most applicable approach for addressing the multiple interventions in this NMA, and its posterior probability provided a ranking of the different treatments [23, 38]. Second, a strict eligibility criterion was formulated before implementing the NMA to ensure the baseline of the included RCTs and reduce the clinical heterogeneity. Third, in addition to the clinical effective rate and left ventricular ejection fraction as primary outcomes, it was pivotal to analyse the 6-minute walk test, left ventricular end-diastolic dimension, heart rate, and cardiac output to assess multiple aspects of the patients' cardiac condition.

Several limitations are worthy of being mentioned. First, only RCTs performed in China were included, which leads to a potential publication bias. The imbalance in the clinical status of the patients between the included RCTs may have affected the results due to patients with various cardiac function classifications and disease durations. Second, the sample size for a few of the outcomes could have been more comprehensive. When calculating continuous outcomes, it was necessary to calculate the data before and after the RCTs. However, several RCTs merely reported the latter, and they could not be included in this NMA. Third, the insufficient methodological information provided for the RCTs restricted the credibility of results and directly led to the low rate of GRADE results in this study. Previous research demonstrated that an RCT that does not apply allocation concealment and blinding to its implementation may overrate the efficacy. Most RCTs did not conceal the allocation or use complete blinding, which resulted in an overestimation of the effects of the included CHIs.

Because of the foregoing shortcomings, we propose several suggestions for future RCTs on CHIs. It is imperative to register in advance and be congruent with the CONSORT standard for RCTs as a way of being retrospective [39]. In an attempt to reveal the objective efficacy of CHIs, researchers should better implement RCTs using allocation concealment and blinding when possible. Although blinding methods are difficult for RCTs of CHIs, it is possible to blind assessors to lower detection bias. Furthermore, clinicians should present dialectical theory in the treatment of DCM, which is a notable feature of traditional Chinese medicine theory. If a syndrome differentiation was determined for patients when diagnosed, meta-analysis researchers could analyse the clinical data from various types of patients and provide specific advice.

## 5. Conclusions

The present NMA compared 6 tonic CHIs in the treatment of DCM and revealed several findings. The results demonstrated that Shenfu injection plus Western medicine exerted a positive effect on improving the overall efficacy of DCM treatment, especially in the clinical effective rate, left ventricular ejection fraction, and 6-minute walk test. Shenmai injection plus Western medicine had a good curative effect in left ventricular end-diastolic dimension and cardiac output. However, it is necessary for clinicians to make diagnostic decisions relying on the efficacy of the CHIs and the patients' situation. Further evidence with larger sample sizes and higher quality is needed to boost the conclusions of this NMA.

## Figures and Tables

**Table 1:** Characteristics of the included randomized controlled trials.

Study ID	Sample size (T/C)	Sex (M/F)	Average age (year)	Disease duration (year)	NYHA(T/C)			Treatment group	Solution of Chis	Control group	Course of treatment (day)	Outcomes
					II	III	IV					
Yang XL 2009	30/30	NR	NR	NR		NR		HQI20 ml + WM	5%GS250 ml	WM	14 d	①
Wu XH 2005	26/26	28/24	E:39.10 ± 17.92 C:41.20 ± 16.81	NR	—	14/15	12/11	HQI30 ml + WM	5%GS250 ml	WM	15 d	①②⑤
Chen DM 2005	60/60	84/36	E:42.5 C:41.7	NR		NR		HQI40 ml + WM	5%GS250 ml	WM	14 d	①
Shang WM 2007	36/36	43/29	59 ± 12	NR				HQI40 ml + WM	5%GS250 ml	WM	14 d	①②
Luo HM 2007	36/32	36/32	E:46 ± 3 C:48 ± 4	E:8.4 ± 0.3 C:8.5 ± 0.2	12/14	14/10	10/8	HQI40 ml + WM	5%GS250 ml	WM	15 d	①⑦
Gao XJ 2010	18/15	24/9	E:42 C:40	E:3–15 C:0.4–15	5/4	7/6	6/5	HQI20 ml + WM	5%GS250 ml/0.9%NS250 ml for diabetic	WM	14 d/course, 2 courses	①②
Lei HF 2011	30/30	60/0	41.5 ± 7.2	NR		NR		HQI20 ml + WM	0.9%NS250 ml	WM	15 d, some patients may be extended to 20∼30 d	①⑦
Liang CC 2001	37/37	42/32	E:56 C55	NR	—	23/14	23/14	SFI20 ml + WM	5%GS100 ml	WM	—	①②④⑥
Que HX 2003	32/28	37/23	E:35–76 C:33–75	E:0.8–15 C:0.8–15	8/7	21/19	3/2	SFI60 ml + WM	5%GS250 ml or 0.9%NS250 ml	WM	14 d	①②⑥
Tang BN 2005	29/29	40/18	E:48.8 C:49.8	NR	1/2	15/17	13/10	SFI60 ml + WM	5%GS250 ml	WM	14 d/course, 2 courses	①②
Yang Y 2009	30/30	42/18	E:42.50 ± 16.81 C:40.52 ± 15.98	NR	—	8/12	22/18	SFI60 ml + WM	5%GS250 ml	WM	14 d	①②⑤⑦
Chen ZG 2009	30/27	42/15	E:21–75 C:20–76	NR	—	20/18	10/9	SFI50 ml + WM	5%GS250 ml/plus insulin for diabetic	WM	14 d	①②④⑤
Lv G 2010	31/30	44/17	E:41.5 ± 10.2 C:40.3 ± 12.5	NR	—	10/11	21/19	SFI50 ml + WM	5%GS250 ml/plus insulin for diabetic	WM	14 d	①②④⑤
Wang CK 2011	50/50	66/34	E:31 ± 9 C:32 ± 9	E:6.1 ± 1.8 C:6.5 ± 1.5	23/21	19/23	8/6	SFI1 ml/(kg·d) + WM	NR	WM	14 d	②③
Nie YJ 2012	38/40	52/26	58.5 ± 10.5	NR	13	45	20	SFI2 ml/(kg·d) + WM	5%GS250 ml	WM	10 d	①②⑤
Yu M 2013	39/40	40/39	40 ± 13	0. 5–12		NR		SFI50 ml + WM	NR	WM	14 d	②③④⑤
Zhang F 2014	40/40	44/36	E:52.1 ± 12.6 C:49.9 ± 13.8	E:6.5 ± 4.6 C:6.2 ± 5.1		NR		SFI50 ml + WM	5%GS250 ml/0.9%NS250 ml	WM	14 d	①
Wang L 2014	42/38	49/31	E:61.8 ± 10.3 C:60.8 ± 9.9	NR	—	12/30	21/17	SFI50 ml + WM	5%GS250 ml	WM	10 d	①②
Qi CH2015	60/60	82/38	E:63.00 C:62.70	NR	—	31/28	29/32	SFI60 ml + WM	5%GS250 ml	WM	14 d	①②⑥
Wu XH 2001	28/28	33/23	E:40.3 ± 15.0 C:41.5 ± 16.8	E:0.1–4.0 C:0.1–4.2	—	16/15	12/13	SI40-60 ml + WM	5%GS250 ml	WM	15 d/course, 2 courses	①②⑤⑦
Zhang YC 2002	50/50	59/41	E:58 ± 15 C:60 ± 16	E:0.5–8.0 C:0.6–8.0	10/10	35/36	5/4	SI60 ml + WM	5%GS250 ml/0.9%NS250 ml	WM	14d	①②⑥
Wang H 2006	30/20	30/20	E:41.0 ± 15.0 C:39.5 ± 13.5	E:0.5–6.0 C:0.4–5.5	11/9	14/9	5/2	SI40-60 ml + WM	5%GS250 ml	WM	14 d/course, 2 courses	①④
Li W 2006	30/22	35/17	E:39.5 C:37.6	E:4.8 C:4.2	13/10	11/8	6/4	SI60 ml + WM	5%GS250 ml/0.9%NS250 ml	WM	14 d	①②④⑥
Wu XL 2009	30/30	42/18	E:45–81 C:41–79	E:0.7–7 C:0.6–8	8/8	16/18	6/4	SI60 ml + WM	5%GS250 ml	WM	14 d	①
Li BH2015	30/30	40/20	53 ± 6	NR		NR		SI50 ml + WM	5%GS500 ml	WM	10 d	①②③④⑦
Shi L 2017	38/38	43/33	E:45.05 ± 7.15 C:46.08 ± 7.12	E: 9.31 ± 3.51 C:9.16 ± 3.37	14/12	15/16	9/10	SI40 ml + WM	5%GS250 ml	WM	14 d/course, 2 courses	①②⑤⑥⑦
Wang NX 2006	15/15	25/5	E:36–59 C:39–58	NR		NR		SMI60 ml + WM	5%GS250 ml/0.9%NS250 ml	WM	15 d	①②
Wang X 2008	30/30	34/26	E:39.6 C:41.2	NR	9/8	15/16	6/6	SMI50 ml + WM	5%GS250 ml	WM	7 d/course, 4 courses	①
Cao Y 2011	111/111	140/82	E:36–59 C:39–58	NR		NR		SMI60 ml + WM	5%GS250 ml	WM	15 d	①
Wang AC 2011	30/30	—	—	NR		NR		SMI100 ml + WM	NR	WM	28 d	①②③⑦
Chen XY 2012	34/34	38/30	42.2	NR	18/19	14/11	2/4	SMI60 ml + WM	5%GS250 ml	WM	14 d	①⑦
Cao L 2012	24/22	31/15	E:42.6 ± 9.8 C:40.1 ± 15.2	NR	—	18/16	6/6	SMI60 ml + WM	5%GS150 ml	WM	14 d	①②⑦
Tian HM 2012	30/30	34/26	E:48.6 ± 5.8 C:49.8 ± 2.5	NR	8/12	14/12	8/6	SMI50 ml + WM	5%GS250 ml	WM	7 d/course, 3 courses	①
Wu JJ 2013	30/30	41/19	—	E:0.3–10 C:0.3–11		NR		SMI60 ml + WM	5%GS250 ml/0.9%NS250 ml for diabetic	WM	14 d	①②⑦
Yan GQ 2014	58/58	62/54	40.6	NR	22/16	28/33	8/9	SMI60 ml + WM	5%GS250 ml	HQI40 ml + WM	15 d	①
Zhao XR 2015	50/50	57/43	E:54.2 ± 4.5 C:54.7 ± 4.2	E: 5.2 ± 1.9 C:5.1 ± 1.5	6/5	25/26	19/19	SMI60 ml + WM	5%GS150 ml	WM	14 d	①②③④⑦
Wang JY 2016	30/30	34/26	E:59.3 ± 8.6 C:60.7 ± 10.2	NR	6/24	10/20	—	SMI100 ml + WM	NR	WM	14 d	①②③⑥
Song CH 2017	50/50	52/48	E:35.25 ± 4.61 C:36.06 ± 4.05	NR	12/10	29/26	9/14	SMI40 ml + WM	5%GS250 ml	WM	14d	①②④⑥⑦
Duan Y 2006	40/42	39/43	E:63.8 ± 4.5 C:59.9 ± 5.2	E:12.6 ± 6.9 C:13.4 ± 7.2	3/4	18/20	19/18	SQFZI250 ml + WM	—	WM	14d	②
Li GK 2014	41/39	44/36	E:67 ± 8 C:66 ± 7	NR	28/25	13/14	—	YQFMI2.6 g + WM	5%GS250 ml	WM	14d	②③④

C, control group; F, female; GS, dextrose solution; HQI, Huangqi injection; M, male; NR, not related; NS, normal saline; SFI, Shenfu injection; SI, Shengmai injection; SMI, Shenmai injection; SQFZI, Shenqi Fuzheng injection; T, treatment group; YQFMI, Yiqifumai injection. Outcomes: ① the clinical effective rate, ② left ventricular ejection fraction, ③ 6-minute walk test, ④ left ventricular end-diastolic dimension, ⑤ heart rate, ⑥ cardiac output, ⑦ ADRs/ADEs.

**Table 2:** Odds ratio/mean difference (95% CIs) of various interventions for all interventions.

Intervention	The clinical effective rate	Left ventricular ejection fraction	6-minute walk test	Left ventricular end-diastolic dimension	Heart rate	Cardiac output
*Huangqi injection* *+* *WM versus*						
Shenfu injection + WM	1.32 (0.63, 2.77)	−3.03 (−14.61, 9.05)	—	—	9.73 (30.64, 51.04)	—
Shengmai injection + WM	1.10 (0.52, 2.31)	0.33 (−11.01, 11.62)	—	—	8.08 (−31.68, 48.92)	—
Shenmai injection + WM	1.14 (0.62, 2.11)	−0.06 (−12.70, 13.15)	—	—	—	—
Shenqi Fuzheng injection + WM	—	−3.07 (−23.08, 16.11)	—	—	—	—
Yiqifumai injection + WM	—	−2.59 (−23.88, 18.61)	—	—	—	—
WM	**0.28 (0.16, 0.48)**	4.35 (−6.35, 15.39)	—	—	2.33 (−36.19, 48.92)	—

*Shenfu injection* *+* *WM versus*						
Shengmai injection + WM	0.83 (0.40, 1.72)	3.43 (−3.04, 9.22)	3.30 (−43.76, 50.75)	−0.82 (−9.73, 8.41)	−1.75 (−19.17, 15.46)	−0.06 (−2.50, 2.49)
Shenmai injection + WM	0.87 (0.44, 1.64)	3.04 (−5.68, 11.62)	23.68 (−60.90, 117.00)	1.22 (−10.09, 12.33)	—	−0.18 (−3.33, 2.85)
Shenqi Fuzheng injection + WM	—	−0.11 (−17.75, 16.76)	—	—	—	—
Yiqifumai injection + WM	—	0.27 (−18.05, 19.07)	25.02 (−47.42, 101.60)	−3.03 (−20.58, 14.69)	—	—
WM	**0.21 (0.12, 0.34)**	**7.43 (2.41, 12.38)**	**50.39 (25.78, 76.33)**	−3.37 (−10.95, 4.39)	−7.55 (−20.73, 5.86)	0.48 (−1.22, 2.51)

*Shengmai injection* *+* *WM versus*						
Shenmai injection + WM	1.05 (0.54, 2.07)	−0.37 (−7.75, 7.38)	20.95 (−71.87, 122.00)	1.99 (−7.90, 11.50)	—	−0.12 (−3.24, 2.78)
Shenqi Fuzheng injection + WM	—	−3.42 (−20.29, 13.08)	—	—	—	—
Yiqifumai injection + WM	—	−3.06 (−20.89, 15.47)	22.42 (−60.02, 105.10)	−2.29 (−18.64, 14.20)	—	—
WM	**0.26 (0.15, 0.43)**	**3.88 (1.10, 8.05)**	**46.43 (5.27, 88.48)**	−2.47 (−7.92, 2.27)	−5.79 (−16.50, 5.00)	0.51 (−1.04, 2.41)

*Shenmai injection* *+* *WM versus*						
Shenqi Fuzheng injection + WM	—	−3.10 (−20.97, 14.20)	—	—	—	—
Yiqifumai injection + WM	—	−2.74 (−20.97, 14.20)	1.80 (−114.00, 109.70)	−4.03 (−22.14, 13.23)	—	—
WM	**0.24 (0.16, 0.37)**	4.36 (−2.47, 11.31)	26.47 (−65.86, 109.60)	−4.53 (−12.86, 3.91)	—	0.65 (−1.62, 3.38)

*Shenqi Fuzheng injection* *+* *WM versus*						
Yiqifumai injection + WM	—	0.60 (−23.54, 24.96)	—	—	—	—
WM	—	7.48 (−8.75, 24.20)	—	—	—	—

*Yiqifumai injection* *+* *WM versus*						
WM	—	7.13 (−10.87, 24.71)	24.37 (−46.95, 95.41)	−0.31 (−16.01, 15.41)	—	24.37 (−46.95, 95.41)

Bold results indicate statistical significance.

**Table 3:** Ranking probability of the various interventions among all interventions.

Intervention	The clinical effective rate		Left ventricular ejection fraction		6-minute walk test		Left ventricular end-diastolic dimension		Heart rate		Cardiac output	
	SUCRA (%)	Rank	SUCRA (%)	Rank	SUCRA (%)	Rank	SUCRA (%)	Rank	SUCRA (%)	Rank	SUCRA (%)	Rank
Huangqi injection + WM	49.0	4	48.1	5	—	—	—	—	37.9	3	—	—
Shenfu injection + WM	78.5	1	71.9	1	75.2	1	60.8	2	70.9	1	54.9	3
Shengmai injection + WM	58.5	3	46.5	6	69.9	2	56.7	3	64	2	60.4	2
Shenmai injection + WM	64.0	2	49.0	4	46.7	3	69.5	1	—	—	60.9	1
Shenqi Fuzheng injection + WM	—	—	62.5	2	—	—	—	—	—	—	—	—
Yiqifumai injection + WM	—	—	59.7	3	44.7	4	39.8	4	—	—	—	—
WM	0.00	5	12.2	7	13.5	5	23.1	5	27.2	4	23.8	4

## Data Availability

The datasets generated during the analysis are not publicly available because the analysis process is the core of the results and cannot be made public. Requests to access the datasets of included studies can be met by checking out them in databases. The study gathered data from Embase, PubMed, the Cochrane Library, the China National Knowledge Infrastructure Database (CNKI), the Wanfang Database, the CQVIP Database (VIP), and the China Biology Medicine disc (SinoMed) database.

## Abbreviations

95% CI: 95% credible interval
ADRs/ADEs: Adverse drug reactions/adverse drug events
CHIs: Chinese herbal injections
DCM: Dilated cardiomyopathy
GRADE: Grading of Recommendations Assessment, Development, and Evaluation
MD: Mean difference
NMA: Network meta-analysis
OR: Odds ratio
RCTs: Randomized controlled trials
SUCRA: The surface under the cumulative ranking
WM: Western medicine.

## Supplementary Materials

Supplementary Materials This file contains five parts, which include items regarding the PRISMA checklist for network meta-analysis and corresponding pages of this study, the search strategy of traditional Chinese medicine injections in Chinese databases and PubMed database, information of included RCTs, details about the product information of CHIs, and the outcome and rate in GRADE of each outcome.
