# Supplementary material for: Investigation on the Efficiency of Tonic Chinese Herbal Injections for Treating Dilated Cardiomyopathy Based on Bayesian Network Meta-Analysis
Source: Evid Based Complement Alternat Med. 2021 Apr 1;2021:8838826. doi: 10.1155/2021/8838826 (PMC8035002; doi:10.1155/2021/8838826)
Supplement: Supplementary Materials — This file contains five parts, which include items regarding the PRISMA checklist for network meta-analysis and corresponding pages of this study, the search strategy of traditional Chinese medicine injections in Chinese databases and PubMed database, information of included RCTs, details about the product information of CHIs, and the outcome and rate in GRADE of each outcome. [file 8838826.f1.doc]

| **Section/Topic** | **#** | **Checklist Item** | **Reported on Page #** |
| --- | --- | --- | --- |
| **TITLE** | | | |
| Title | 1 | Identify the report as a systematic review incorporating a network meta-analysis (or related form of  meta-analysis). | 1 |
| **ABSTRACT** | | | |
| Structured summary | 2 | Provide a structured summary including, as applicable: Background: main objectives; Methods: data sources; study eligibility criteria, participants, and interventions; study appraisal; and synthesis methods, such as network meta-analysis. Results: number of studies and participants identified; summary estimates with corresponding confidence/credible intervals; treatment rankings may also be discussed. Authors may choose to summarize pairwise comparisons against a chosen treatment included in their analyses for brevity. Discussion/Conclusions: limitations; conclusions and implications of findings. Other: primary source of funding; systematic review registration number with registry name. | 2 |
| **INTRODUCTION** | | | |
| Rationale | 3 | Describe the rationale for the review in the context of what is already known, including mention of why a network meta-analysis has been conducted. | 3 |
| Objectives | 4 | Provide an explicit statement of questions being addressed with reference to participants, interventions, comparisons, outcomes, and study design (PICOS). | 3-4 |
| **METHODS** | | | |
| Protocol and registration | 5 | Indicate if a review protocol exists and if and where it can be accessed (e.g., Web address), and, if available, provide registration information including registration number. | / |
| Eligibility criteria | 6 | Specify study characteristics (e.g., PICOS, length of follow-up) and report characteristics (e.g., years considered, language, publication status) used as criteria for eligibility, giving rationale. Clearly describe eligible treatments included in the treatment network, and note whether any have been clustered or merged into the same node (with justification). | 4-5 |
| Information sources | 7 | Describe all information sources (e.g., databases with dates of coverage, contact with study authors to identify additional studies) in the search and date last searched. | 4 |
| Search | 8 | Present full electronic search strategy for at least one database, including any limits used, such that it could be repeated. | 4 |
| Study selection | 9 | State the process for selecting studies (i.e., screening, eligibility, included in systematic review, and, if applicable, included in the meta-analysis). | 5 |
| Data collection process | 10 | Describe method of data extraction from reports (e.g., piloted forms, independently, in duplicate) and any processes for obtaining and confirming data from investigators. | 5 |
| Data items | 11 | List and define all variables for which data were sought (e.g., PICOS, funding sources) and any assumptions and simplifications made. | 5 |

Page 2 of 2

| **Section/Topic** | **#** | **Checklist Item** | **Reported on Page #** |
| --- | --- | --- | --- |
| Geometry of the network | 12 | Describe methods used to explore the geometry of the treatment network under study and potential biases related to it. This should include how the evidence base has been graphically summarized for presentation, and what characteristics were compiled and used to describe the evidence base to readers. | 6 |
| Risk of bias within individual studies | 13 | Describe methods used for assessing risk of bias of individual studies (including specification of whether this was done at the study or outcome level), and how this information is to be used in any data synthesis. | 5-6 |
| Summary measures | 14 | State the principal summary measures (e.g., risk ratio, difference in means). Also describe the use of additional summary measures assessed, such as treatment rankings and surface under the cumulative ranking curve (SUCRA) values, as well as modified approaches used to present summary findings from meta-analyses. | 6 |
| Planned methods of analysis | 15 | Describe the methods of handling data and combining results of studies for each network meta-analysis. This should include, but not be limited to: Handling of multigroup trials; Selection of variance structure; Selection of prior distributions in Bayesian analyses; and Assessment of model fit. | 6 |
| Assessment of inconsistency | 16 | Describe the statistical methods used to evaluate the agreement of direct and indirect evidence in the treatment network(s) studied. Describe efforts taken to address its presence when found. | 6 |
| Risk of bias across studies | 17 | Specify any assessment of risk of bias that may affect the cumulative evidence (e.g., publication bias, selective reporting within studies) | 6 |
| Additional analyses | 18 | Describe methods of additional analyses if done, indicating which were prespecified. This may include, but not be limited to, the following: Sensitivity or subgroup analyses; Meta-regression analyses; Alternative formulations of the treatment network; and Use of alternative prior distributions for Bayesian analyses (if applicable). | 6 |
| **RESULTS** | | | |
| Study selection | 19 | Give numbers of studies screened, assessed for eligibility, and included in the review, with reasons for exclusions at each stage, ideally with a flow diagram. | 6-7 |
| Presentation of network structure | 20 | Provide a network graph of the included studies to enable visualization of the geometry of the treatment network. | 9-10 |
| Summary of network geometry | 21 | Provide a brief overview of characteristics of the treatment network. This may include commentary on the abundance of trials and randomized patients for the different interventions and pairwise comparisons in the network, gaps of evidence in the treatment network, and potential biases reflected by the network structure. | 9-10 |
| Study characteristics | 22 | For each study, present characteristics for which data were extracted (e.g., study size, PICOS, follow-up period) and provide the citations. | 8-9 |
| Risk of bias within studies | 23 | Present data on risk of bias of each study and, if available, any outcome level assessment. | 9 |
| Results of individual studies | 24 | For all outcomes considered (benefits or harms), present, for each study: 1) simple summary data for each intervention group, and 2) effect estimates and confidence intervals. Modified approaches may be needed to deal with information from larger networks. | 13-19 |
| **Section/Topic** | **#** | **Checklist Item** | **Reported on Page #** |
| Synthesis of results | 25 | Present results of each meta-analysis done, including confidence/credible intervals. In larger networks, authors may focus on comparisons versus a particular comparator (e.g., placebo or standard care), with full findings presented in an appendix. League tables and forest plots may be considered to summarize pairwise comparisons. If additional summary measures were explored (such as treatment rankings), these should also be presented. | 21 |
| Exploration for inconsistency | 26 | Describe results from investigations of inconsistency. This may include such information as measures of model fit to compare consistency and inconsistency models, P values from statistical tests, or summary of inconsistency estimates from different parts of the treatment network. | 21-22 |
| Risk of bias across studies | 27 | Present results of any assessment of risk of bias across studies for the evidence base being studied. | 21 |
| Results of additional analyses | 28 | Give results of additional analyses, if done (e.g., sensitivity or subgroup analyses, meta-regression  analyses, alternative network geometries studied, alternative choice of prior distributions for  Bayesian analyses, and so forth). | 19 |
| **DISCUSSION** | | | |
| Summary of evidence | 29 | Summarize the main findings, including the strength of evidence for each main outcome; consider their relevance to key groups (e.g., health care providers, researchers, and policymakers). | 22-23 |
| Limitations | 30 | Discuss limitations at study and outcome level (e.g., risk of bias), and at review level (e.g., incomplete retrieval of identified research, reporting bias). Comment on the validity of the assumptions, such as transitivity and consistency. Comment on any concerns regarding network geometry (e.g., avoidance of certain comparisons). | 24 |
| Conclusions | 31 | Provide a general interpretation of the results in the context of other evidence, and implications for future research. | 25 |
| **FUNDING** | | | |
| Funding | 32 | Describe sources of funding for the systematic review and other support (e.g., supply of data); role of funders for the systematic review. This should also include information regarding whether funding has been received from manufacturers of treatments in the network and/or whether some of the authors are content experts with professional conflicts of interest that could affect use of treatments in the network. | 26 |

*From:* Moher D, Liberati A, Tetzlaff J, Altman DG, The PRISMA Group (2009). Preferred Reporting Items for Systematic Reviews and Meta-Analyses: The PRISMA Statement. PLoS Med 6(6): e1000097. doi:10.1371/journal.pmed1000097

For more information, visit: **www.prisma-statement.org**.

Page 3 of 3

**Search strategy**

**1. Search strategy of Chinese herbal injections**

| Names of Chinese Herbal injections | English Searching Words | Chinese Searching Words |
| --- | --- | --- |
| Huangqi injection | Huangqi OR Astragalus | 黄芪注射剂 OR 黄芪注射液 OR 注射用黄芪 |
| Shenfu injection | Shenfu | 参附注射剂 OR 参附注射液 OR 注射用参附 |
| Shengmai injection | Shengmai | 生脉注射剂 OR 生脉注射液 OR 注射用生脉 |
| Shenmai injection | Shenmai | 参麦注射剂 OR 参麦注射液 OR 注射用参麦 |
| Shenqi Fuzheng injection | Shenqi Fuzheng | 参芪扶正注射剂 OR 参芪扶正注射液 OR 注射用参芪扶正 OR 参芪扶正 |
| Yiqifumai injection | Yiqifumai | 益气复脉注射剂 OR 益气复脉注射液 OR 注射用益气复脉 |

**2. Pubmed:**

#1 randomized controlled trial [Publication Type]

#2 controlled clinical trial [Publication Type]

#3 randomized [Title/Abstract]

#4 placebo [Title/Abstract]

#5 clinical trials as topic [MeSH Major Topic]

#6 randomly [Title/Abstract]

#7 trial [Title]

#8 #1 OR #2 OR #3 OR #4 OR #5 OR #6 OR #7

#9 animals [MeSH] NOT humans [MeSH]

#10 #8 NOT #9

#11 huangqi [Title/Abstract]

#12 astragalus [Title/Abstract]

#13 shengfu [Title/Abstract]

#14 shengmai [Title/Abstract]

#15 shenmai [Title/Abstract]

#16 shenqi fuzheng [Title/Abstract]

#17 yiqifumai [Title/Abstract]

#18 #11 OR #12 OR #13 OR #14 OR #15 OR #16 OR #17

#19 dilated cardiomyopathy [MeSH Terms]

#20 dilated cardiomyopathy [Title/Abstract]

#21 #19 OR #20

#22 #10 AND #18 AND #21

**3. Cochrane Library:**

#1 randomized controlled trial, pt

#2 controlled clinical trial, pt

#3 randomized, ti,ab,kw

#4 placebo, ti,ab

#5 clinical trials as topic, mesh

#6 randomly, ti,ab

#7 trial, ti

#8 #1 OR #2 OR #3 OR #4 OR #5 OR #6 OR #7

#9 huangqi: ti,ab,kw

#10 astragalus: ti,ab,kw

#11 shengfu: ti,ab,kw

#12 shengmai: ti,ab,kw

#13 shenmai: ti,ab,kw

#14 shenqi fuzheng : ti,ab,kw

#15 yiqifumai: ti,ab,kw

#16 #9 OR #10 OR #11 OR #12 OR #13 OR #14 OR #15

#17 dilated cardiomyopathy: MeSH

#18 dilated cardiomyopathy: ti,ab,kw

#19 #17 OR #18

#20 #8 AND #16 AND #19

**4. EMBASE:**

#1 randomized controlled trial/exp

#2 controlled clinical trial

#3 randomized, ti,ab

#4 placebo, ti,ab

#5 clinical trial(topic)

#6 randomly, ti,ab

#7 trial, ti

#8 #1 OR #2 OR #3 OR #4 OR #5 OR #6 OR #7

#9 #8 AND [humans]/lim

#10 huangqi

#11 astragalus

#12 shengfu

#13 shengmai

#14 shenmai

#15 shenqi fuzheng

#16 yiqifumai

#17 #10 OR #11 OR #12 OR #13 OR #14 OR #15 OR #16

#18 dilated cardiomyopathy/exp

#19 dilated cardiomyopathy /ti,ab,kw

#20 #18 OR #19

#21 #9 AND #17 AND #20

**Information about the included randomized controlled trials**

1. Cao L, Zhang P, Li L. Efficacy of shenmai injection in the treatment of dilated cardiomyopathy. Chin J Mod Drug (Chin) 2012; 06(18):88-88.
2. Cao Y, Gao ZY. Clinical observation of 111 cases of dilated cardiomyopathy treated with shengmai injection. Shaanxi Med. J. (Chin) 2011; 40(1): 119-119.
3. Chen DM. Clinical study on 60 cases of dilated cardiomyopathy treated with astragalus injection. Chin. J. Misdiagn. (Chin) 2005; (11): 2060-2061.
4. Chen XY. Observation on 34 Cases of Dilated Cardiomyopathy Treated with Shenmai Injection. Zhejiang Clin. Med. J. (Chin) 2012; 47(02): 153.
5. Chen ZG, Li HJ, Zhang SR. Evaluation of shenfu injection in patients with dilated cardiomyopathy. Med. Innov. China (Chin) 2009; 6(35): 3-4.
6. Duan Y, Zhang YL. Clinical observation of dilated cardiomyopathy treated with shenqi fuzheng injection. Chin. Commun. Doct. (Chin) 2006; (17): 67-68.
7. Gao XJ. Clinical observation of 18 cases of dilated cardiomyopathy combined with Chinese and western medicine. Yunnan J. Tradit. Chin. Med. Mater. Med. (Chin) 2010; 31(05): 29.
8. Lei HF. Clinical observation of 30 cases of dilated cardiomyopathy treated with astragalus injection. Jilin Med. J. (Chin) 2011; 32(27): 5706-5707.
9. Li BH, Wang XY, Jin WD, Shi JJ. Study on the effect of shengmai injection in the treatment of decompensated heart failure caused by dilated cardiomyopathy. Contemp. Med. Forum (Chin) 2015; 13(23): 137-138.
10. Li GK. Study on the treatment of dilated cardiomyopathy treated with yiqifumai injection and shenmai injection. Pract. Clin. J. Integr. Tradit. Chin. West. Med. (Chin) 2014; 14(3):76-77.
11. Li W. Clinical study of shengmai injection in the treatment of dilated cardiomyopathy. [dissertation]. [China]: Hubei College of Traditional Chinese Medicine. 2006.
12. Liang CC, Zheng YH. Curative effect of Chinese and western medicine on dilated cardiomyopathy. Liaoning J. Tradit. Chin. Med. (Chin) 2001; (02): 107.
13. Luo HM. Observation of the effect of astragalus injection on dilated cardiomyopathy. Guangxi J. Tradit. Chin. Med. (Chin) 2007; (02): 22-23.
14. Lv G. Analysis of 31 cases of dilated cardiomyopathy treated by shenfu injection. Chin. Prac. Med. (Chin) 2010; 05(20): 175-176.
15. Nie YJ, Song HH, Qi CX. Effect of shenfu injection in the treatment of dilated cardiomyopathy with heart failure clinical observation of 38 cases. China Med. Pharm. (Chin) 2012; 02(20): 64-65.
16. Qi CH, Qi L. Clinical observation of shenfu injection in the treatment of dilated cardiomyopathy with congestive heart failure. J. Emerg. Syndromes Tradit. Chin. Med. (Chin) 2015; 24(06): 1096-1098.
17. Que HX, Guo JH. Clinical observation of dilated cardiomyopathy treated with shenfu injection. Fujian Med. J. (Chin) 2003; 25(2): 125-126.

# Shang WM, Ma MF. Clinical observation of 36 cases of dilated cardiomyopathy treated with astragalus injection. Cent. Plains Med. J. (Chin) 2007; 34(23): 80.

1. Shi L, Wang XF, Zhou JX. Effects of shengmai injection on the efficacy and heart rate variability of patients with dilated cardiomyopathy and heart failure. Pharmacol. Clin. Chin. Mater. Med. (Chin) 2017; (03): 179-182.
2. Song CH. Effect of shenmai injection combined with western medicine on dilated cardiomyopathy combined with heart failure. Mod. J. Integr. Tradit. Chin. West. (Chin) 2017; (11): 95-97.
3. Tang BN, Wang HQ. Clinical observation of 29 cases of dilated cardiomyopathy combined with Chinese and western medicine. Hebei J. Tradit. Chin. Med. (Chin) 2005; (12): 938.
4. Tian HM, Hu YJ, Wang SL. Clinical observation of dilated cardiomyopathy combined with Chinese and western medicine. Chin. J. Ethnomed. Ethnopharm. (Chin) 2012; 21(23): 83-83.
5. Wang AC. Ginseng injection in the treatment of dilated cardiomyopathy heart failure qi and Yin deficiency syndrome in clinical research. [China]: Shandong University of Traditional Chinese Medicine. 2011.
6. Wang CK, Wang YH, Zhao B, Xie ZS. Study about efficacy of shenfu injection in the treatment of dilated cardiomyopathy with congestive heart failure. Chin. Heart J. (Chin) 2011; 23(05): 703-704.
7. Wang H. Clinical observation of pulse-activating injection in treating dilated cardiomyopathy. J. Henan Univ. Chin. Med. (Chin) 2006; (04):29-30.
8. Wang JY. Clinical effect of shenmai injection in the treatment of dilated cardiomyopathy combined with chronic heart failure and its effect on quality of life. Zhejiang J. Integr. Tradit. Chin. West. (Chin) 2016; 26(3): 236-238.
9. Wang L, Huang ZH, Guo LT, Zhang YB. Clinical effects of Shenfu Injection in the treatment of patients with dilated cardiomyopathy combined with chronic systolic heart failure. Chin. J. Tradit. Chin. Med. Pharm. (Chin) 2014; 29(10): 3348-3350.
10. Wang NX, Gao X. Efficacy of shenmai injection in the treatment of heart failure with dilated cardiomyopathy. J. Pract. Med. Tech. (Chin) 2006; 13(8): 1293-1293.
11. Wang X, Zhou WJ. Clinical observation of 30 cases of dilated cardiomyopathy combined with Chinese and western medicine. Zhejiang Clin. Med. J. (Chin) 2008; 10(8): 1104-1105.
12. Wu JJ, Qie J. Efficacy of shenmai injection in the treatment of dilated cardiomyopathy. Public Med. Forum Mag. (Chin) 2013; 17(26): 3415-3416.
13. Wu XH, Chen KW. Clinical observation of 26 cases of dilated cardiomyopathy treated with astragalus injection. Yunnan J. Tradit. Chin. Med. Mater. Med. (Chin) 2005; (01): 13.
14. Wu XH, Jiao JL, Shi RJ. Clinical observation of 28 cases of dilated cardiomyopathy combined with shengmai injection. Chin. J. Integr. Med. (Chin) 2001; (03): 230.
15. Wu XL. Effect of shengmai injection in the treatment of dilated cardiomyopathy. China Foreign Med. Treat. (Chin) 2009; 28(28): 190-190,192.
16. Yan GQ. Study on the treatment of dilated cardiomyopathy treated with huangqi injection and shenmai injection. Neimonggu J. Tradit. Chin. (Chin) 2014; 33(29): 3-3.
17. Yang XL, Zhang ZX. Study about efficacy of astragalus injection in the treatment of dilated cardiomyopathy with congestive heart failure. Clin. Med. (Chin) 1998; (09): 38.
18. Yang Y, Wu XH. Clinical observation of 30 cases of dilated cardiomyopathy treated with shenfu injection. Yunnan J. Tradit. Chin. Med. Mater. Med. (Chin) 2009; 30(9): 26-27.
19. Yu M, Lv SM, Liu ZL, Fang RJ. Study about efficacy of shenfu injection combined with trimetazidine in the treatment of dilated cardiomyopathy. Chin. J. Tradit. Med. Sci. Technol. (Chin) 2013; 20(6): 642-643.
20. Zhang F, Ren KH, Chen YL. Effect of Shenfu injection on cardiac function and ventricular remodeling in patients with dilated cardiomyopathy associated with heart failure. Chin. J. Clin. Pharmacol. (Chin) 2014; 30(06): 478-480.
21. Zhang YC, Chen RM, Zhao MH, Lv BJ, Rong HZ. Effect of shengmai injection on hemodynamics in patients with dilated cardiomyopathy. Chin. J. Integr. Med. (Chin) 2002; (04): 277-279.
22. Zhao XR. Effect discussion of Shenmai injection in the treatment of dilated cardiomyopathy combined with heart failure. China Mod. Med. (Chin) 2015; 22(32): 148-150.

**Detailed information on Chinese herbal injections**

| **Chinese herbal injection** | **Source** | **Raw material** | **Phytochemical compositions** | **Chemical composition criteria** | **Therapeutic claims in TCM** | **Indications** | **Quality control reported? (Y/N)** | **Chemical analysis reported? (Y/N)** |
| --- | --- | --- | --- | --- | --- | --- | --- | --- |
| Ciwujia injection | Wandashan Pharmaceutical in Heilongjiang province | *ACANTHOPANACIS SENTICOSI RADIX ET RHIZOMA SEU CAULIS* (Manyprickle Acanthopanax) | Eleutheroside, Syringoside, Isofraxidin and et al. | The **total flavonoids** contained in this product are calculated as anhydrous Rutin (C_27_H_30_O_16_) and should be 90.0-110.0% of the labeled amount.  The labeled amount is: 20 ml of the injection contains 100 mg of total flavonoids; 100 ml of the injection contains 300 mg of total flavonoids; and 250 ml of the injection contains 500 mg of total flavonoids. | Moderate reinforcing liver and kidney, replenishing essence, toning up bones. | coronary heart disease, angor pectoris with neurasthenia, transient ischemic attack, cerebral arteriosclerosis, cerebral infarction caused by deficiency of the liver and kidney essence , etc. | Y - National Food and Drug Administration National Drug Standards | N |
| Dazhuhongjingtian injection | Tonghua Yusheng Pharmaceutical Co., Ltd. | *RHODIOLAE CRENULATAE RADIX ET RHIZOMA* (Rhodiola Rosea) | Salidroside, Rhodiosin, Rhodionin, Herbacetin, Kaempferol, Quercetin and et al. | This product contains 1 mg of **total sugar** in anhydrous glucose (C_6_H_12_O_6_), not less than 20.0mg.  Not less than 3.5mg per 1ml of **Salidroside** (C_14_H_20_O_7_), not less than 0.30mg of **Tyrosol** (C_8_H_10_O_2_). | Activating blood and dissolving stasis. | Stable angina pectoris and others caused by insufficient heart blood with the symptoms of stabbing pain and colic pain in chest, chest distress, palpitation, and thread pulse, etc. | Y - National Food and Drug Administration National Drug Standards | N |
| Huangqi injection | CHENGDU DIAO PHARMACEUTICAL GROUP Co., Ltd./ CHIATAI QINGCHUNBAO Pharmaceutical Co., Ltd./ Shanghai Fuda Pharmaceutical Drug manufacturing Co., Ltd. | *ASTRAGALI RADIX* (Mikvetch Root) | Astragaloside, Isoastragaloside, Acetylastragaloside, Astragaline, Benzofiiranoidlignan and et al. | Each 1ml of Huangqi injection contains not less than 0.08mg of **Astragaloside IV** (C_41_H_68_O_14_). | *Qi* tonifying and body strengthening resistance, heart nourishment, spleen fortification and dampness removal. | Insufficiency of the heart-*qi* and blood stasis syndrome of viral myocarditis, cardiac insufficiency and et al.; hepatitis with spleen deficiency and dampness syndrome, etc. | Y - National Food and Drug Administration National Drug Standards | N |
| Shenfu injection | Yaan Three Nine Pharmaceutical Co., Ltd. | *GINSENG RADIX ET RHIZOMA RUBRA* (Red Ginseng)*, ACONm LATERALIS RADIX PRAEPARAIA* (Prepared Common Monkshood Daughter Root) | Ginsenoside, Benzoyldeoxyaconitine, Benzoylhypaconitine, Benzoylaconitine, Benzoylmesaconitine and et al. | Each 1ml containing **total Ginsenoside Saponins** is not less than 0.5mg based on Ginsenoside Rb_1_ (C_54_H_92_O_23_). | Revives *yang* for resuscitation, *qi* tonifying and exhaustion prevention. | Desertion syndrome caused by excessive *yang*-*qi* insufficiency (infectious, haemorrhagic and fluid loss shock); *yang* or *qi* deficiencies with palpitations, cough, stomach ache, diarrhoea, rheumatism, etc. | Y - National Food and Drug Administration National Drug Standards | N |
| Shengmai injection | Jiangsu Suzhong Construction Group Co., Ltd./ Changshu LEI YUN SHANG Pharmaceutical Co., Ltd./ Shanxi TAIHANG Pharmaceutical Co., Ltd. | *GINSENG RADIX ET RHIZOMA RUBRA* (Red Ginseng)*, OPHIOPOGONIS RADIX* (Dawarf Lilyturf Tuber)*, SCHISANDRAE CHINENSIS FRUCTUS* (Chinese Magnoliavine Fruit) | Ginsenoside, Panaxatriol, Ophiopogonone, Methylophiopogonanone, Shikimic Acid and et al. | Each 1ml contains **Ginsenoside Rg1** not less than 0.08mg, containing **Ginsenoside Re** not less than 0.04mg. | Nourishing *qi* and *yin*, exhaustion prevention. | Palpitations, hard breathing, cold limbs and other symptoms caused by *qi* and *yin* insufficiencies, myocardial infarction, cardiogenic shock, cardiogenic shock and others with abovementioned symptoms, etc. | Y - National Food and Drug Administration National Drug Standards | N |
| Shenmai injection | CHIATAI QINGCHUNBAO Pharmaceutical Co., Ltd./ West China Medical University Pharmaceutical Factory/ Sichuan Sanjing Shenghe Pharmaceutical Ltd./ Yaan Three Nine Pharmaceutical Co., Ltd./ Hebei Shenwei Pharmaceutical Co., Ltd. | *GINSENG RADIX ET RHIZOMA RUBRA* (Red Ginseng)*,* 4g  *OPHIOPOGONIS RADIX* (Dawarf Lilyturf Tuber) 4g | Ginsenoside, Ophiopogonin, Ophiopogon Polysaccharide and et al. | The **total saponin** per 1ml is not less than 0.80 mg based on Ginsenoside Re (C_48_H_82_O_18_). | *Qi* tonifyng, exhaustion prevention, *yin* nourishment, body fluid generation, pulse activation. | Shock, coronary heart disease, viral myocarditis, chronic pulmonary heart disease and neutropenia with *qi* and *yin* deficiencies; improves immune function of patients with tumours, combined with chemotherapy to enhance curative effects and to reduce toxic and side effects, etc. | Y - National Food and Drug Administration National Drug Standards | N |
| Shenqi Fuzheng injection | LIVZON Pharmaceutical Group Co., Ltd. | *ASTRAGALI RADIX* (Mikvetch Root)*, CODONOPSIS RADIX* (Tangshen) | Astragaloside I, Astragaloside II, Astragaloside III, Astragaloside IV, Lobetyolin, Formononetin and et al. | Not less than 13.0 mg per 1 ml of **total solids**.  The **total saponin** per 1 ml is not less than 0.12 mg based on Astragaloside IV (C_41_H_68_O_14_).  Each 1 ml containing **Huangqi** is not less than 0.004 mg based on Astragaloside (C_41_H_68_O_14_).  The total **sugar** contained per 1 ml is not less than 3.5 mg based on anhydrous glucose (C6H12O6).  This product contains **sodium chloride (NaCl)** should be 98.0% -104.0% of the indicated amount. | *Qi* toning and *yin* nourishment*.* | Fatigue, lack of strength, vertigo caused by asthenia of pulmonosplenic *qi*; auxiliary treatment of the above symptoms for lung cancer and gastric cancer, etc. | Y - National Food and Drug Administration National Drug Standards | N |

**References**

1. Cao, L. M., Zhao, X., Liu, T. T., Tang, M., Li, Q., Pan, Y., et al. (2017). Simultaneous determination of five components in school of pharnacy, Shenfu injection with HPLC method. Pharm Clin Chin Mater Med. 7, 22-24.
2. Cao, M. M., Gao, Y. Y., Ma, Y. X., and Li, J. (2015). Research progress on the chemical composition of astragalus membranaceus and its protective effect on myocardial ischemia reperfusion injury. Inf Tradit Chin Med. 32, 120-123.
3. Fan, H. X. (2014). Pharmacokinetic study of the major active components of acanthopanax senticoccus. University of Jinan.
4. Gu, H. Y., Zhang, S. Y., Huang, W. H., Liu, X. H., Wang, Y., Fan, C. L., et al. (2013). Chemical constituents of shenqi fuzheng injection. Chin Tradit Pat Med. 35, 1494-1499.
5. Huang, J., Shao, Q., Xiang, X. H., Ge, Z. W., and Fan, X. H. (2014). Identification of phenylpropanoids in ciwujia injection by HPLC-MS. *China J Chin Mater Med.* 39, 2513-2520.
6. Li, N., Huang, X., Wang, B., Zhang, J. Y., Wang, H. Y., Dong, T. X., et al. (2017). Identification of chemical constituents of ophiopogon japonicus in shenmai injection. Chin Tradit Pat Med. 39, 2340-2344.
7. Liu, W. S. (2011). Study on HPLC fingerprint of red ginseng and shenfu injection. Shenyang Pharmaceutical University.
8. Shu, J., and Yang, S. B. (2016). Simultaneous determination of six components in shenqi fuzheng injection by HPLC. China Pharm. 27, 4295-4297.
9. Su, P., Lv, S. F., Fan, X. M, Liang, Q. L., Wang, Y. M., and Luo, G. A. (2011). Protective effect of shenmai injection and its effective components on H2 O2 induced myocardial cell injury. Chin Tradit Pat Med. 33, 2150-2154.
10. Wang, Q., Liu, Y., Zheng., X. W., Yu, J. D., Dai, Z., Lu, J., et al. (2012). Study on homoisoflavones in shengmai injection. Chin Pharm J. 47, 1539-1542.
11. Wang, Q., Zheng., X. W., Yu, J. D., Liu, Y., Dai, Z., Lu, J., et al. (2012). Study on saponins in shengmai injection. China J Chin Mater Med. 18, 1731-1734.
12. Wu, J. G., Dong, L., Chen, H. J., and Xu, X. P. (2017). Determination of shikimic acid in shengmai injection by HPLC. Chin Measur Test Technol. 43, 41-44.
13. Yang, R. J. (2012). Study on chemical constituents of shenfu injection. Jilin University.
14. Zhang, S. Y., Fan, C. L., Wang, L., Liu, X. H., Sun, X. W., and Ye, W. C. (2011). Chemical constituents of shenqi fuzheng injection. Chin Tradit Pat Med. 33, 1743-1748. 3. Zhao, L. (2017). Determination of the active ingredients of rhodiola extract and the effect of hypoxia on the pharmacokinetics. Capital Institute of Medicine.
15. Zhao, L., Qi, T., Hui, B. K., Xu, P. X., and Xue, M. (2016). Quantification of six active ingredients in rhodiola extract by UPLC-MS/MS. J Int Pharm Res. 43, 975-979.
16. Zhao, Y. D. (2016). Chemical composition of astragalus injection and its systematic evaluation on cerebral infarction. Beijing University of Traditional Chinese Medicine.
17. The outcome and rate in GRADE of the clinical effective rate.

| Comparison | Direct evidence | | Indirect evidence | | Network meta-analysis | |
| --- | --- | --- | --- | --- | --- | --- |
|  | Odds ratio  (95% confidence interval) | Quality of evidence | Odds ratio  (95% confidence interval) | Quality of evidence | Odds ratio  (95% confidence interval) | Quality of evidence |
| HQI+WM vs. SFI+WM | - | - | 1.32 (0.63, 2.77) | Low^b^ | 1.32 (0.63, 2.77) | Very low^4^ |
| HQI+WM vs. SI+WM | - | - | 1.10 (0.52, 2.31) | Very low^b^ | 1.10 (0.52, 2.31) | Very low^4^ |
| HQI+WM vs. SMI+WM | 2.48 (0.80, 7.66) | Moderate*^,1^ | 0.82 (0.39, 1.73) | Low^b^ | 1.14 (0.62, 2.11) | **Low^4^** |
| HQI+WM vs. WM | 4.22 (2.36, 7.53) | Low^1,3^ | 0.71 (0.20, 2.90) | Low^b^ | 0.28 (0.16, 0.48) | **Low** |
| SFI+WM vs. SI+WM | - | - | 0.83 (0.40, 1.72) | Very low^b^ | 0.83 (0.40, 1.72) | Very low^4^ |
| SFI+WM vs. SMI+WM | - | - | 0.87 (0.44, 1.64) | Low^b^ | 0.87 (0.44, 1.64) | Very low^4^ |
| SFI+WM vs. WM | 4.56 (2.84, 7.33) | Low^1,3^ | Not estimable** | Not estimable** | 0.21 (0.12, 0.34) | **Low** |
| SI+WM vs. SMI+WM | - | - | 1.05 (0.54, 2.07) | Very low^b^ | 1.05 (0.54, 2.07) | Very low^4^ |
| SI+WM vs. WM | 3.76 (2.29, 6.17) | Very Low^1,2,3^ | Not estimable** | Not estimable** | 0.26 (0.15, 0.43) | Very low |
| SMI+WM vs. WM | 3.49 (2.34, 5.21) | Low^1,3^ | 0.09 (0.02, 0.33) | Low^b^ | 0.24 (0.16, 0.37) | **Low** |

^1^Limitations (Risk of bias). ^2^Inconsistency. ^3^Publication bias. ^4^Impricision. *There is only 1 RCT in this comparison. The estimate of inconsistency and publication bias ae not applicable. **Cannot be estimated because the drug was not connected in a loop in the evidence network. ^a^Contributing direct evidence of moderate quality. ^b^Contributing direct evidence of low or very low quality

1. The outcome and rate in GRADE of Left ventricular ejection fraction.

| Comparison | Direct evidence | | Indirect evidence | | Network meta-analysis | |
| --- | --- | --- | --- | --- | --- | --- |
|  | Mean deviation  (95% confidence interval) | Quality of evidence | Mean deviation  (95% confidence interval) | Quality of evidence | Mean deviation  (95% confidence interval) | Quality of evidence |
| HQI+WM vs SFI+WM | - | - | -3.03 (-14.61, 9.05) | Very Low^b^ | -3.03 (-14.61, 9.05) | Very Low^4^ |
| HQI+WM vs SI+WM | - | - | 0.33 (-11.01, 11.62) | Very Low^b^ | 0.33 (-11.01, 11.62) | Very Low^4^ |
| HQI+WM vs SMI+WM | - | - | -0.06 (-12.70, 13.15) | Very Low^b^ | -0.06 (-12.70, 13.15) | Very Low^4^ |
| HQI+WM vs SQFZI+WM | - | - | -3.07 (-23.08, 16.11) | Low^b^ | -3.07 (-23.08, 16.11) | Very Low^4^ |
| HQI+WM vs YQFMI+WM | - | - | -2.59 (-23.88, 18.61) | Low^b^ | -2.59 (-23.88, 18.61) | Very Low^4^ |
| HQI+WM vs WM | 4.18 (2.07, 6.29) | Low^1,3^ | Not estimable** | Not estimable** | 4.35 (-6.35, 15.39) | Very Low^4^ |
| SFI+WM vs SI+WM | - | - | 3.43 (-3.04, 9.22) | Very Low^b^ | 3.43 (-3.04, 9.22) | Very Low^4^ |
| SFI+WM vs SMI+WM | - | - | 3.04 (-5.68, 11.62) | Very Low^b^ | 3.04 (-5.68, 11.62) | Very Low^4^ |
| SFI+WM vs SQFZI+WM | - | - | -0.11 (-17.75, 16.76) | Very Low^b^ | -0.11 (-17.75, 16.76) | Very Low^4^ |
| SFI+WM vs YQFMI+WM | - | - | 0.27 (-18.05, 19.07) | Very Low^b^ | 0.27 (-18.05, 19.07) | Very Low^4^ |
| SFI+WM vs WM | 7.44 (5.16, 9.71) | Very Low^1,2,3^ | Not estimable** | Not estimable** | 7.43 (2.41, 12.38) | Very Low |
| SI+WM vs SMI+WM | - | - | -0.37 (-7.75, 7.38) | Very Low^b^ | -0.37 (-7.75, 7.38) | Very Low^4^ |
| SI+WM vs SQFZI+WM | - | - | -3.42 (-20.29, 13.08) | Very Low^b^ | -3.42 (-20.29, 13.08) | Very Low^4^ |
| SI+WM vs YQFMI+WM | - | - | -3.06 (-20.89, 15.47) | Very Low^b^ | -3.06 (-20.89, 15.47) | Very Low^4^ |
| SI+WM vs WM | 6.22 (3.32, 9.11) | Very Low^1,2,3^ | Not estimable** | Not estimable** | 3.88 (1.10, 8.05) | Very Low |
| SMI+WM vs SQFZI+WM | - | - | -3.10 (-20.97, 14.20) | Very Low^b^ | -3.10 (-20.97, 14.20) | Very Low^4^ |
| SMI+WM vs YQFMI+WM | - | - | -2.74 (-21.76, 16.68) | Very Low^b^ | -2.74 (-21.76, 16.68) | Very Low^4^ |
| SMI+WM vs WM | 4.87 (2.57, 7.18) | Very Low^1,2,3^ | Not estimable** | Not estimable** | 4.36 (-2.47, 11.31) | Very Low^4^ |
| SQFZI+WM vs YQFMI+WM | - | - | 0.60 (-23.54, 24.96) | Low^b^ | 0.60 (-23.54, 24.96) | Very Low^4^ |
| SQFZI+WM vs WM | 8.00 (5.48, 10.52) | Low*# | Not estimable** | Not estimable** | 7.48 (-8.75, 24.20) | Very Low^4^ |
| YQFMI+WM vs WM | 7.16 (4.32, 10.00) | Moderate*^,1^ | Not estimable** | Not estimable** | 7.13 (-10.87, 24.71) | **Low^4^** |

^1^Limitations (Risk of bias). ^2^Inconsistency. ^3^Publication bias. ^4^Impricision. #Severe limitations (risk of bias). *There is only 1 RCT in this comparison. The estimate of inconsistency and publication bias ae not applicable. **Cannot be estimated because the drug was not connected in a loop in the evidence network. ^a^Contributing direct evidence of moderate quality. ^b^Contributing direct evidence of low or very low quality

1. The outcome and rate in GRADE of 6-minute walk test

| Comparison | Direct evidence | | Indirect evidence | | Network meta-analysis | |
| --- | --- | --- | --- | --- | --- | --- |
|  | Mean deviation  (95% confidence interval) | Quality of evidence | Mean deviation  (95% confidence interval) | Quality of evidence | Mean deviation  (95% confidence interval) | Quality of evidence |
| SFI+WM vs SI+WM | - | - | 3.30 (-43.76, 50.75) | Moderate^a^ | 3.30 (-43.76, 50.75) | **Low**^4^ |
| SFI+WM vs SMI+WM | - | - | 23.68 (-60.90, 117.00) | Low^b^ | 23.68 (-60.90, 117.00) | Very Low^4^ |
| SFI+WM vs YQFMI+WM | - | - | 25.02 (-47.42, 101.60) | Moderate^a^ | 25.02 (-47.42, 101.60) | **Low**^4^ |
| SFI+WM vs WM | 53.89 (50.04, 57.74) | Moderate^1^ | Not estimable** | Not estimable** | 50.39 (25.78, 75.33) | **Moderate** |
| SI+WM vs SMI+WM | - | - | 20.95 (-71.87, 122.00) | Low^b^ | 20.95 (-71.87, 122.00) | Very Low^4^ |
| SI+WM vs YQFMI+WM | - | - | 22.42 (-60.02, 105.10) | Moderate^a^ | 22.42 (-60.02, 105.10) | **Low^4^** |
| SI+WM vs WM | 50.20 (42.48, 57.92) | Moderate*^,1^ | Not estimable** | Not estimable** | 46.43 (5.27, 88.48) | **Moderate** |
| SMI+WM vs YQFMI+WM | - | - | 1.80 (-114.00, 109.70) | Low^b^ | 1.80 (-114.00, 109.70) | Very Low^4^ |
| SMI+WM vs WM | 48.35 (32.53, 64.17) | Low^1,3^ | Not estimable** | Not estimable** | 26.47 (-65.86, 109.60) | Very Low^4^ |
| YQFMI+WM vs WM | 23.49 (10.27, 36.71) | Moderate*^,1^ | Not estimable** | Not estimable** | 24.37 (-46.95, 95.41) | **Low^4^** |

^1^Limitations (Risk of bias). ^2^Inconsistency. ^3^Publication bias. ^4^Impricision. *There is only 1 RCT in this comparison. The estimate of inconsistency and publication bias ae not applicable. **Cannot be estimated because the drug was not connected in a loop in the evidence network. ^a^Contributing direct evidence of moderate quality. ^b^Contributing direct evidence of low or very low quality

1. The outcome and rate in GRADE of left ventricular end-diastolic dimension

| Comparison | Direct evidence | | Indirect evidence | | Network meta-analysis | |
| --- | --- | --- | --- | --- | --- | --- |
|  | Mean deviation  (95% confidence interval) | Quality of evidence | Mean deviation  (95% confidence interval) | Quality of evidence | Mean deviation  (95% confidence interval) | Quality of evidence |
| SFI+WM vs SI+WM | - | - | -0.82 (-9.73, 8.41) | Very low^b^ | -0.82 (-9.73, 8.41) | Very low^4^ |
| SFI+WM vs SMI+WM | - | - | 1.22 (-10.09, 12.33) | Moderate^a^ | 1.22 (-10.09, 12.33) | **Low**^4^ |
| SFI+WM vs YQFMI+WM | - | - | -3.03 (-20.58, 14.69) | Moderate^a^ | -3.03 (-20.58, 14.69) | **Low^4^** |
| SFI+WM vs WM | -3.22 (-4.41, -2.02) | Moderate^1^ | Not estimable** | Not estimable** | -3.37 (-10.95, 4.39) | **Low**^4^ |
| SI+WM vs SMI+WM | - | - | 1.99 (-7.90, 11.50) | Very low^b^ | 1.99 (-7.90, 11.50) | Very low^4^ |
| SI+WM vs YQFMI+WM | - | - | -2.29 (-18.64, 14.2) | Very low^b^ | -2.29 (-18.64, 14.2) | Very low^4^ |
| SI+WM vs WM | -3.76 (-6.36, -1.16) | Very low^1,2,3^ | Not estimable** | Not estimable** | -2.47 (-7.92, 2.27) | Very low^4^ |
| SMI+WM vs YQFMI+WM | - | - | -4.03 (-22.14, 13.23) | Moderate^a^ | -4.03 (-22.14, 13.23) | **Low^4^** |
| SMI+WM vs WM | -4.59 (-5.66, -3.53) | Moderate^1^ | Not estimable** | Not estimable** | -4.53 (-12.86, 3.91) | **Low**^4^ |
| YQFMI+WM vs WM | -0.20 (-2.56, 2.16) | Moderate*^,1^ | Not estimable** | Not estimable** | -0.31 (-16.01, 15.41) | **Low**^4^ |

^1^Limitations (Risk of bias). ^2^Inconsistency. ^3^Publication bias. ^4^Impricision. *There is only 1 RCT in this comparison. The estimate of inconsistency and publication bias ae not applicable. **Cannot be estimated because the drug was not connected in a loop in the evidence network. ^a^Contributing direct evidence of moderate quality. ^b^Contributing direct evidence of low or very low quality
